# Proton MRS of cervical cancer at 7 T

**DOI:** 10.1002/nbm.4015

**Published:** 2018-10-30

**Authors:** C.S. Arteaga de Castro, J.P. Hoogendam, I.M.L. van Kalleveen, A.J.E. Raaijmakers, R.P. Zweemer, R.H.M. Verheijen, P.R. Luijten, W.B. Veldhuis, D.W.J. Klomp

**Affiliations:** ^1^ Department of Radiology UMC Utrecht The Netherlands; ^2^ Department of Gynecological Oncology UMC Utrecht Cancer Center The Netherlands; ^3^ Department of Radiology the Netherlands Cancer Institute Amsterdam The Netherlands

**Keywords:** 7 T, cervical cancer, fatty acids, FIGO stage, MRS, tumor grade

## Abstract

The differentiation grade of cervical cancer is histologically assessed by examining biopsies or surgical specimens.

MRS is a highly sensitive technique that images tissue metabolism and can be used to increase the specificity of tissue characterization in a non‐invasive manner. We aim to explore the feasibility of using in vivo ^1^H‐MRS at 7 T in women with cervical cancer to study tissue fatty acid composition.

10 women with histologically proven Stage IB1‐IIB cervical cancer were scanned with a whole‐body 7 T MR system with a multi‐transmit system and an internal receive only monopole antenna. A STEAM sequence was used to obtain ^1^H‐MRS data. Fatty acid resonances were fitted with Lorentzian curves and the 2.1 ppm/1.3 ppm ratios were calculated.

^1^H‐MRS data showed fatty acid signals resonating at 2.1 ppm, 1.9 ppm, 1.5 ppm, 1.3 ppm and 0.9 ppm. Mean 2.1/1.3 ppm ratios were 0.019 ± 0.01, 0.021 ± 0.006, 0.12 ± 0.089 and 0.39 ± 0.27 for normal, Grade I, Grade II and Grade III groups respectively. Poorly differentiated tumor tissue (Grade III) showed elevated fatty acid ratios when compared with the well differentiated tumor (Grade I) or normal tissue.

^1^H‐MRS in cervical cancer at 7 T is feasible and individual fatty acid signals were detected. In addition, poorly differentiated tumors show more fatty acid unsaturation. The 2.1 ppm/1.3 ppm ratio has potential for tumor characterization in a non‐invasive manner for uterine cervical cancer.

**Abbreviations used**
AC: adenocarcinoma
ERA: endorectal antenna
FIGO: International Federation of Gynecology and Obstetrics
FOV: field of view
GUI: graphical user interface
Rx: receive only
SCC: squamous cell carcinoma
SNR: signal to noise ratio
STEAM: stimulated echo acquisition mode
SV: single voxel
T2w: *T*_2_ weighted
*T*_E_: echo time
*T*_R_: repetition time

## 1 INTRODUCTION

Tumor tissue characterization relies on histologic information, typically obtained by biopsy, which may be prone to sampling errors ranging from 10.6% to 43%.^1^, ^2^, ^3^, ^4^, ^5^ This means that, due to the intratumor heterogeneity, the biopsy location often mismatches the location of the tumor or highest grade as identified on post‐surgical histological specimens. This can be improved using non‐invasive methods such as MRS that can preoperatively measure tissue metabolism, which has shown to be beneficial in early tissue differentiation.^6^, ^7^, ^8^ Alterations in fatty acid metabolism have been observed with MRS in diverse tumor and tissue types including uterine cervix,^9^, ^10^, ^11^ suggesting rapidly growing tumors.^12^, ^13^ Studies with ^14^C have corroborated that elevated fatty acids arise from novo‐synthesis.^9^, ^10^ In the uterine cervix, alterations in fatty acid metabolism (the *α*‐carboxyl (2.24 ppm) and *α*‐olefin (2.02 ppm), observed as one peak resonating at 2.1 ppm, methylene at 1.3 ppm and methyl at 0.9 ppm) have been used to discriminate pre‐invasive and invasive tumors.^11^, ^14^, ^15^, ^16^, ^17^ In addition, the methylene fatty acid resonance at 1.3 ppm has been used to differentiate between cancer and healthy control groups.^18^ An important limitation of these studies was the data acquisition at 1.5 T, where fatty acid resonance frequencies overlap.

Ultra‐high magnetic field strengths such as 7 T offer an increased signal to noise ratio (SNR) and spectral resolution, which can be exploited for individual detection of metabolites that overlap at lower fields. This allows exploration of fatty acid profiles and the possibility to detect otherwise overlapping fatty acids or metabolites at ultra‐high field. However, the strong non‐uniformities found at these field strengths, due to wavelength shortening, make the use of a body coil (based on loop coils) inefficient. Instead, external antennas with more penetrating *B*_1_ power can be used. The use of an additional receive only (Rx) internal antenna can further boost SNR in the region of interest (i.e. cervix).^19^, ^20^, ^21^, ^22^, ^23^, ^24^ In the particular case of uterine cervix imaging, internal Rx coils can be introduced transvaginally. However, given their limited diameter, transvaginal coils restrict the imaging to the forward view of the coil. In the case of larger tumors, transvaginal coils may have a suboptimal performance. Endorectal monopole antennas have an improved far‐field performance and can cover larger regions.^25^, ^26^, ^27^

We propose to exploit the SNR available at 7 T in combination with an Rx internal antenna to explore the feasibility of tumor characterization in uterine cervix by the fatty acid profile measured with ^1^H MRS.

## 2 MATERIALS AND METHODS/EXPERIMENT

**Figure 1:**
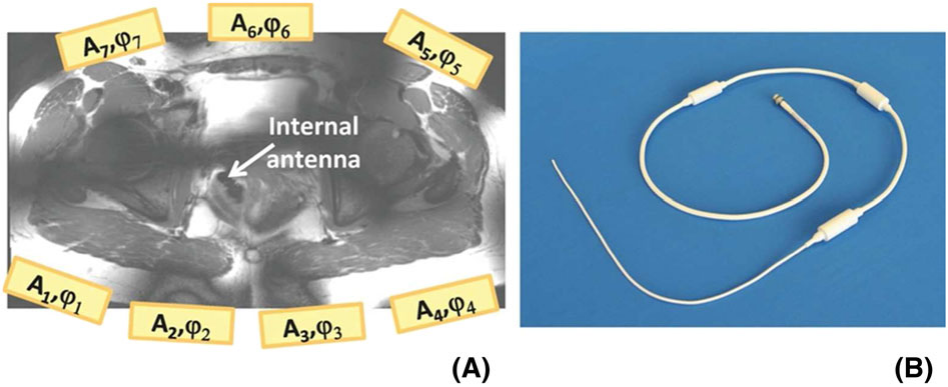
A, Schematic view of the external antenna setup around the body. The location of the 4.7 mm thick internal antenna used for reception (B) is shown with the white arrow

This prospective study was approved by the institutional review board (clinicaltrials.gov: NCT02083848). Ten women with histologically proven Stage IB1‐IIB2 cervical cancer were included in this study after giving informed consent. Patients were excluded when definitive therapy had already been initiated. They all underwent a 1.5 T MRI (standard) clinical examination in addition to the 7 T MR protocol. Afterwards, all patients underwent radical therapy and a final histology report was available. In addition, five healthy female volunteers were scanned after giving signed informed consent. The coil setup used for the MR examination is schematically shown in Figure 1, and consisted of seven (or eight in the volunteers' case) external fractionated dipole antennas (MRCoils, Zaltbommel, The Netherlands)^28^ that were positioned around the pelvis of the subject for transmission and reception. A single 4.7 mm thick endorectally placed internal monopole antenna (Machnet, BV, Roden, The Netherlands) (Figure 1B) was used for reception only^29^ for patient scanning. The performance and safety of this endorectal antenna (ERA) has already been established and published by van Kalleveen et al.^25^ The ERA was embedded in a 4.7 mm transparent catheter, which provided electrical isolation and rigidity for better insertion. The antenna was sterilized, covered with a disposable sterile ultrasound cover and smeared with K‐Y lubricant jelly (© Johnson & Johnson, New Brunswick, NJ, US) before insertion. No volunteer agreed to the insertion of the ERA. To compensate for the SNR loss when the ERA is not present during volunteer scanning, the same fractionated dipole transceiver antennas included two integrated Rx loops per channel,^30^, ^31^ resulting in 16 additional Rx channels, which were interfaced to a 16‐channel receiver interface box (Philips Medical Systems, Cleveland, OH, USA). All antennas were interfaced to an eight‐channel multi‐transmit system on a 7 T whole‐body MR system (Philips Medical Systems).

Four of the external antennas were positioned on the MR table and aligned to the dorsal pelvis of the patient, when lying in a supine position. The remaining three or four transceivers were positioned around the ventral pelvis. The center of the external setup was positioned at the level of the pubic symphysis to obtain the maximal *B*_1_^+^ in the cervix. To homogenize and remove any possible signal voids due to deconstructing interference, *B*_1_^+^ (i.e. transmit) phase shimming in the region of interest^32^ was performed. To achieve this, a *T*_1_ weighted gradient echo (2D GE, 3.5° FA, *T*_E_/*T*_R_ = 1.68/30 ms, 252 × 400 × 10 mm field of view (FOV), 3.91 × 3.82 voxel size, eight repetitions, 25 s total scan duration) was acquired, which was loaded on a MATLAB‐based (©MATLAB version 9 R2015b) *B*_1_ shimming graphical user interface (GUI). The GUI optimized the combination of transmit phases based on numerical optimization in a drawn ROI, such as to obtain a homogeneous *B*_1_^+^. These optimized *B*_1_^+^ phases were used for all subsequent scan protocols. *B*_0_ shimming was performed prior any anatomic protocol, in the whole volume.

High resolution *T*_2_ weighted (T2w) images (multi‐slice SE, TSE factor 16, *T*_E_/*T*_R_ = 70/7000 ms, 250 × 4000 × 59 mm FOV, 0.8 × 0.81 mm resolution, 3 mm slice thickness, 15 slices) were acquired in the transverse, sagittal and oblique (to the cervix) planes for anatomy localization. This T2w MRI was used for the planning of the MRS measurement. Single‐voxel (SV) and CSI measurements were obtained with a stimulated echo acquisition mode (STEAM) sequence (repetition/echo times *T*_R_/*T*_E SV,CSI_ = 1400/36–75, 10 ms, varying voxel size from 20 to 50 mm^3^ or 30 × 30 mm^2^ FOV with 5 × 5 × 5 mm^3^ voxels, for the SV or the CSI case respectively, 192 signal averages and 16 phase cycles for the SV case, 2048 acquired points, 4000 acquisition bandwidth). The STEAM sequence was chosen in this study as it only requires low *B*_1_ amplitudes and can reach shorter *T*_E_ times. In the SV MRS acquisitions, the voxels were located such as to include at least 80% of the tumor. A VAPOR (variable power and optimized relaxation delays)^33^ scheme with a fixed 150 Hz bandwidth was used for water suppression in all cases. The CSI grid was positioned in the tumor in all cases.

The MRS acquisitions were Hanning filtered and zero phased. Resonance frequencies were assigned based on the fatty acid resonances previously found in the uterine cervix by Mahon et al. and Hamilton et al. and according to the fatty acids measured at 7 T.^15^, ^34^, ^35^ The SNR ratio was calculated for all spectra using the amplitude of the 1.3 ppm peak and the noise in the spectrum where no peaks are observed. All spectra were fitted in the LCModel based software NMRWizard (version 2012‐10‐09 by Robin A. de Graaf, MRRC, Yale University). Seven resonance frequencies were fitted in NMRWizard, namely 3.2 ppm, 3.0 ppm, 2.1 ppm (approximated from the methylene protons alpha to C=C and COO, 2.04 and 2.25 ppm respectively), 1.9 ppm, 1.5 ppm, 1.3 ppm and 0.9 ppm, with Lorentzian curves, 50 iterations and a 10–100 Hz linewidth range. Baselines were also fitted with the same approach. Fitted peak integrals were used to calculate the fatty acid ratios.

## 3 RESULTS

Median age for this patient population was 38.6 years old (25–66 years). None of the patients reported any discomfort when placing or removing the internal antenna. ^1^H MRS was successful in all 10 patients. A compilation of the medical and histological findings for the scanned patients is shown in Table 1. Seven patients had histologically‐proven adenocarcinoma (AC) and three had squamous cell carcinoma (SCC). Two patients had well differentiated (Grade I) tumors, three had Grade II and five patients poorly differentiated (Grade III) ones. Two patients (Cases 1 and 3) underwent conization, which revealed a higher stage (i.e. IB1), though the residual tumor after this procedure was not visually detectable on the T2w 7 T MRI. Tumor median diameter measured from MRI was 41 cm (25–80 cm).

**Table 1:** Patient information with successful spectroscopy results: histology, FIGO stages, tumor grade, maximum tumor diameter and lipid ratios

Patient number	Histology type	FIGO stage	Pathology grading	Max. tumor diameter (mm)	Lipid ratio 2.1/1.3 ppm
1	AC	IB1	I	not MR visible	0.027
2	AC	IB1	II	25	0.030
3	AC	IB1	II	not MR visible	0.093
4	AC	IB1	III	30	0.480
5	AC	IB2	III	60	0.671
6	SCC	IB2	III	80	0.027
7	AC	IB2	III	60	0.036
8	SCC	IB2	III	70	0.101
9	SCC	IB2	II	100	0.242
10	AC	IIB	I	37	0.014

Median age for the volunteer group was 27.5 years old (24–35 years). None of the volunteers had any previous history of uterine cervix disease. Therefore, all these cases were considered healthy.

High resolution T2w images were obtained successfully in all patients and volunteers for anatomy localization and for ^1^H MRS planning.

**Figure 2:**
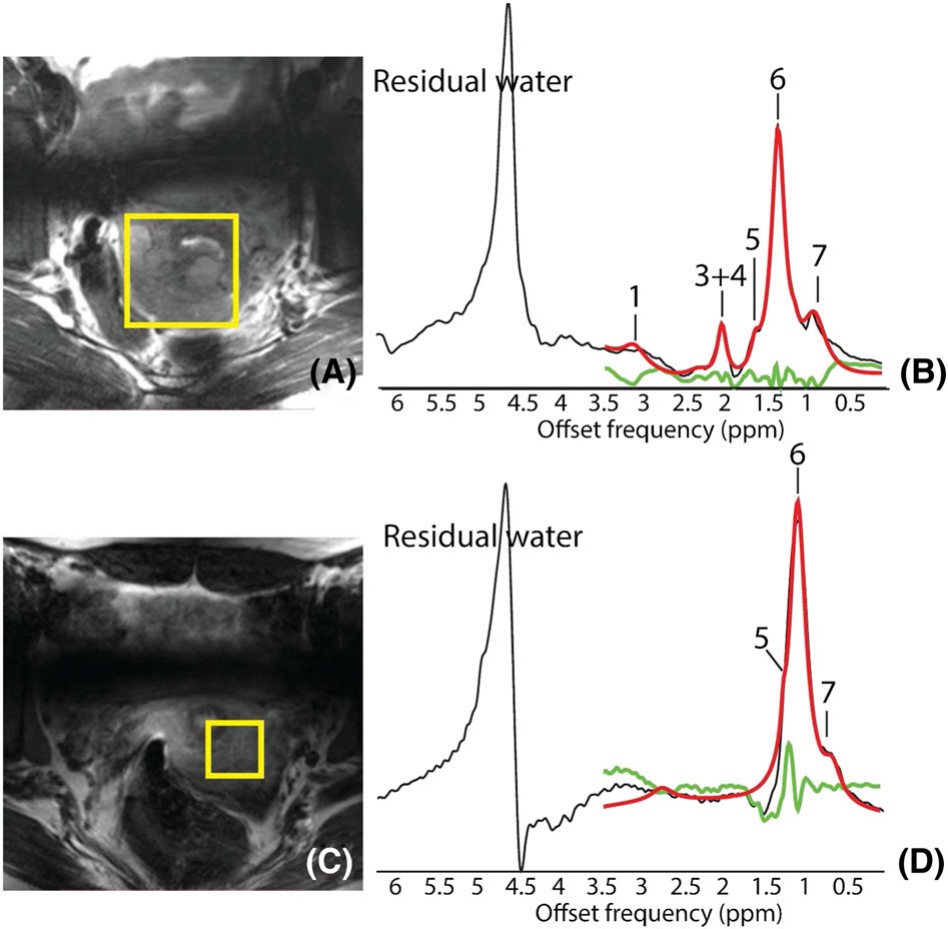
A, C, Zoomed‐in T2w images of the cervix of two histologically‐proven AC cases, with MR visible (FIGO IB2, Grade III) and tumor not visible in MR (FIGO IB1, Grade II). Images show the SV localization for the STEAM acquisitions (B, D) in yellow. Metabolites observed and fitted are labeled as 1, choline, 3, 2.1 ppm, 4, 1.9 ppm, 5, 1.5 ppm, 6, 1.3 ppm, and 7, 0.9 ppm. Overlapped in red is the total fit, in gray the individual fits and in green the residual. Notice the absence of the resonances around 1.9 ppm in D

The resulting spectra obtained with the internal antenna at 7 T had sufficient SNR (99 on average) to detect metabolite (including choline and creatine) and fatty acid signals. The SNR of the MRS acquired in volunteers was 54 on average, which corresponds to 55% of the SNR in patients. The fatty acids resonating at 2.1 ppm and at 1.3 ppm were present in all data sets. Therefore, the 2.1 ppm over 1.3 ppm ratio was calculated for all groups. In addition, the measured baselines were strongly influenced by first order phase artifacts, due to sub‐optimal *B*_0_ shimming and therefore residual water signals. However, the fitting algorithm was able to fit the baselines. Average 2.1 ppm/1.3 ppm fatty acid ratios found were 0.019 ± 0.010, 0.021 ± 0.006, 0.12 ± 0.089 and 0.39 ± 0.270 (a.u.) for the normal, Grade I, Grade II and Grade III groups respectively. These ratios are increased in poorly differentiated tumors, but this is not statistically significant. Figure 2 shows the T2w images with the STEAM localization planning (yellow box) for two patients with histologically proven AC, International Federation of Gynecology and Obstetrics (FIGO) Stage IB2, Grade III and tumor size 60 cm (Figure 2A and 2B) and Stage IB1 with Grade II and a microscopic tumor that was not observed with MRI (Figure 2C and 2D) respectively. The 60 mm tumor was also visible in the MRI at this spatial resolution. Both fitted spectra show the total fitted spectrum in red, the individual fits in gray and the residual in green. Figure 2C and 2D also shows low choline levels. In addition, the fatty acids resonating at 0.9 ppm, 1.1 ppm (as a shoulder‐peak of the 1.3 ppm peak), 1.3 ppm, 1.5 ppm and 2.1 ppm are present in both cases. Only the resonance of the peak located at 1.9 ppm was not observed in a case with no detectable tumor on MR. In addition, the patient with Stage IB2 and Grade III has a higher 2.1 ppm/1.3 ppm (0.67) fatty acid ratio than the patient with Stage IB1, Grade II and no detected tumor (0.093).

**Figure 3:**
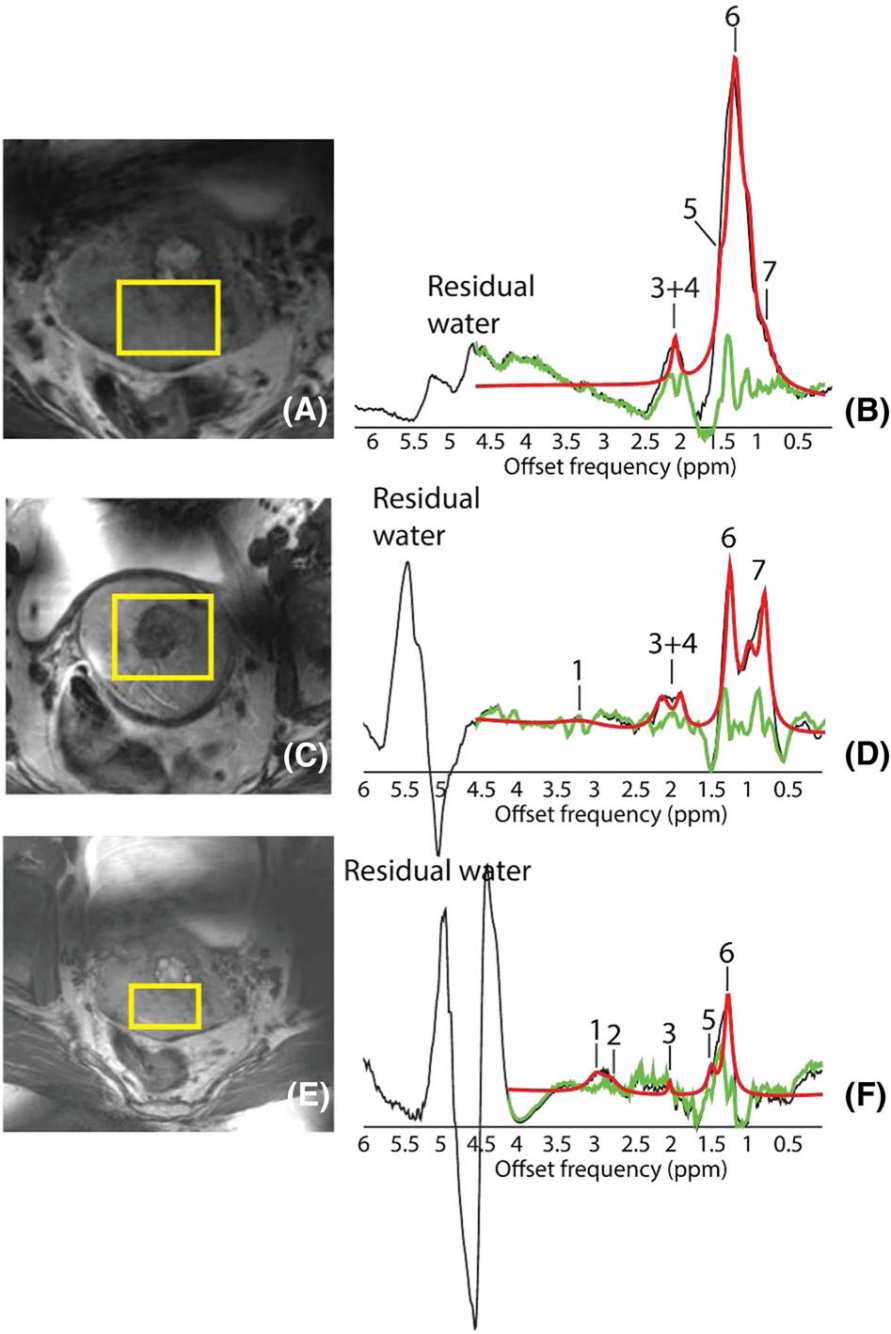
Zoomed‐in T2w images are shown for three different patients with SCC and Grades III, II and III respectively. Volume localization for the STEAM acquisition planning is shown in yellow. Two spectra (D, F) had detectable (black) and fitted (red and gray) levels of choline as well as fatty acids. Metabolites observed and fitted are labeled as 1, choline, 2, creatine, 3, 2.1 ppm, 4, 1.9 ppm, 5, 1.5 ppm, 6, 1.3 ppm, 7, 1.1 ppm, and 8, 0.9 ppm. Overlapped in red is the total fit, in gray the individual fits and in green the residual

Figure 3 shows T2w MRI (A, C, E) of the three cases where choline and creatine were fitted in addition to the fatty acid signals. The spectra in Figure 3B, 3D and 3F correspond to histology‐proven SCC, FIGO Stages IB2, IIB and IB2 and Grades III, II and III respectively. All tumors were detected during diagnosis (maximum diameter 80, 100 and 70 mm respectively) and observed in the MRI for these last cases. In addition, the 2.1 ppm/1.3 ppm ratios were 0.02, 0.27 and 0.10.

**Figure 4:**
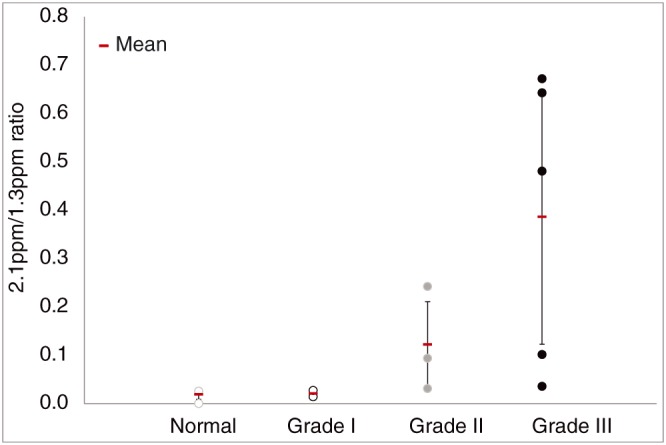
21 ppm/1.3 ppm calculated fatty acid ratios versus the tumor grade. Average values show a trend towards more unsaturated fatty acids in poorly differentiated tumors. However, the standard deviations are still large and calculated ratios overlap between groups

Figure 4 shows the plot of the calculated fatty acid 2.1 ppm over 1.3 ppm ratio versus the corresponding grade group. From this plot we can observe that the average fatty acid chain becomes less saturated in poorly differentiated tumors (Grade III) when compared with well differentiated (Grade I) or normal cervix. The ranges of the 2.1 ppm/1.3 ppm ratio of the different groups overlap.

## 4 DISCUSSION

In vivo ^1^H MRS of uterine cervical carcinomas at 7 T to measure fatty acid profiles has been shown to be feasible for the first time. We have found that the use of an internal antenna in combination with an external array of radiating antennas provides sufficient SNR for MRI and STEAM MRS acquisitions at 7 T. The inclusion of internal antennas to enhance SNR in MRI has been already explored at lower magnetic field strengths to image the uterine cervix, with positive results,^15^, ^19^, ^20^ and has been validated in this pilot study at the field of 7 T. The positive effect of this internal monopole antenna has been recently investigated at our institution for the same patient population.^27^ In particular, the quality of the T2w MRI obtained for all patients was found to be superior to the T2w MRI routinely acquired at 1.5 T.^27^

The MRS results found in this study agree with previous published MRS studies at lower magnetic field strengths, where mainly fatty acid signals were observed in uterine cervical ACs.^11^, ^14^, ^15^, ^16^, ^17^, ^18^, ^36^ The fatty acid oxidation pathway in cancer cells has been recently given more attention and proven to be as important in cancer metabolism as the Warburg effect. Tumor cells prefer the fatty acid oxidation pathway as a source of energy, which can come from either external or newly formed (i.e. from novo‐synthesis) fatty acids, which are oxidized and stored as lipid droplets in the tissue.^12^, ^13^ Therefore, it seems reasonable to characterize the lipid composition in cancer and its relationship to disease, if any, with ^1^H MRS. The presented exploratory study in uterine cervical cancers has shown a trend towards an increase in accumulated fatty acids in poorly differentiated tumors. In particular, the ratio of two fatty acid groups, namely the combination of the *α*‐carboxyl and *α*‐olefinic observed at about 2.1 ppm over the methyl fatty acid resonating at 1.3 ppm, was used here, as these were the most prominent peaks present in all tumors. Despite the small patient population in this pilot study, the 2.1 ppm/1.3 ppm fatty acid ratios showed a trend towards more unsaturated fatty acids in poorly differentiated tumors. Literature has showed that the tissue fatty acid profile can be characterized to differentiate healthy from diseased tissue, for tumor staging and for determination of aggressiveness. He et al. showed differences in the polyunsaturated fatty acid content in breast cancer tissue, compared with the healthy contra part.^37^ Finley et al. observed in serums that a higher aggressiveness in prostate cancer tissue presented elevated peri‐prostatic adipose tissue.^38^ Fatty acid content differences in non‐alcoholic liver disease, which is the precursor of liver cancer, have also been found between the different stages of the disease.^39^, ^40^ All these points stress the relevance of fatty acid profiling in diseased tissue.

The increased spectral resolution available at 7 T enabled depiction of fatty acid peaks that overlap at lower fields. Choline and creatine were detected and fitted in tumor grades II and III.

A limitation of using the methylene 1.3 ppm peak for ratio calculation is that it overlaps with the methyl resonance of lactate, which also appears at 1.3 ppm and is typically present in tumors.^41^, ^42^, ^43^ Therefore, the fitting of this fatty acid resonance can be somewhat overestimated. Moreover, the presence of microscopic intra‐ and extra‐cellular lipids could cause a resonance shift due to their differences in bulk magnetic susceptibility, which could explain the 1.1 ppm shoulder on the 1.3 ppm resonance. Some of these effects could be overcome using editing techniques such as simple editing or double quantum coherence schemes.^44^, ^45^

Although the STEAM sequence has been used extensively in vivo at different magnetic field strengths in many different in vivo investigations, it still suffers from low SNR due to the necessity of using strong dephasing gradients between three 90° RF pulses, which lead to signal loss. This was somewhat compensated in our study by the inclusion of the internal Rx antenna in patients and the additional receivers in the volunteers. In addition, the STEAM RF pulses are sensitive to *B*_1_ non‐uniformities. Therefore, in extensive 2D or 3D CSI volumes the effect of the RF pulses over the whole volume could vary, particularly at higher field strengths, which may lead to incorrect metabolite concentrations and phase artifacts. The calculation of metabolite ratios corrects for these differences. In addition, the poor profiles of the STEAM pulses can introduce spurious chemical shift artifacts, mainly from water in the surrounding tissues, even when water suppression schemes are included. Optimization of the MRS sequence for future studies would be favorable.

## 5 CONCLUSION

We were able to obtain lipid profiles in cervical carcinomas with ^1^H MRS at 7 T. For the first time, it was observed that the 2.1 ppm/1.3 ppm fatty acid ratio might be associated with tumor grade in cervical cancer, as seen by the increased unsaturation in poorly differentiated tumors. Therefore, this ratio may have the potential of characterizing tumor grade in a non‐invasive manner to aid clinical diagnostics.

## FUNDING INFORMATION

STW Technology Foundation (grant no 10822).
